# Estimating PM_2.5_ Concentrations in Xi'an City Using a Generalized Additive Model with Multi-Source Monitoring Data

**DOI:** 10.1371/journal.pone.0142149

**Published:** 2015-11-05

**Authors:** Yong-Ze Song, Hong-Lei Yang, Jun-Huan Peng, Yi-Rong Song, Qian Sun, Yuan Li

**Affiliations:** 1 School of Land Science and Technology, China University of Geosciences, Beijing, China; 2 Department of Geological Engineering, Qinghai University, Xining, Qinghai Province, China; 3 School of Water Resources and Environment, China University of Geosciences, Beijing, China; 4 School of Geophysics and Information Technology, China University of Geosciences, Beijing, China; The Ohio State University, UNITED STATES

## Abstract

Particulate matter with an aerodynamic diameter <2.5 μm (PM_2.5_) represents a severe environmental problem and is of negative impact on human health. Xi'an City, with a population of 6.5 million, is among the highest concentrations of PM_2.5_ in China. In 2013, in total, there were 191 days in Xi’an City on which PM_2.5_ concentrations were greater than 100 μg/m^3^. Recently, a few studies have explored the potential causes of high PM_2.5_ concentration using remote sensing data such as the MODIS aerosol optical thickness (AOT) product. Linear regression is a commonly used method to find statistical relationships among PM_2.5_ concentrations and other pollutants, including CO, NO_2_, SO_2_, and O_3_, which can be indicative of emission sources. The relationships of these variables, however, are usually complicated and non-linear. Therefore, a generalized additive model (GAM) is used to estimate the statistical relationships between potential variables and PM_2.5_ concentrations. This model contains linear functions of SO_2_ and CO, univariate smoothing non-linear functions of NO_2_, O_3_, AOT and temperature, and bivariate smoothing non-linear functions of location and wind variables. The model can explain 69.50% of PM_2.5_ concentrations, with R^2^ = 0.691, which improves the result of a stepwise linear regression (R^2^ = 0.582) by 18.73%. The two most significant variables, CO concentration and AOT, represent 20.65% and 19.54% of the deviance, respectively, while the three other gas-phase concentrations, SO_2_, NO_2_, and O_3_ account for 10.88% of the total deviance. These results show that in Xi'an City, the traffic and other industrial emissions are the primary source of PM_2.5_. Temperature, location, and wind variables also non-linearly related with PM_2.5_.

## Introduction

Particulate matter with an aerodynamic diameter less than 2.5 μm, termed PM_2.5_, is a mixture of various gaseous and particulate components, and the primary source of PM2.5 is believed to be anthropogenic emission such as combustion process [1]. The monitoring and study of PM_2.5_ is important because PM_2.5_ can negatively impact human health [2, 3]. Because of its small size, PM_2.5_ has a long lifetime in the atmosphere and can be transmitted great distance, leading to remote deposit locations in the respiratory tract of human beings. It can readily infiltrate people's lungs, reach alveoli and hardly be eliminated from the body [4]. The primary source of toxicity is the heavy metal elements and toxic organic matters attached on PM_2.5_, which could affect the normal functions of human body, delay the human development, and even cause heart disease and cancer after entering the body [5–7]. Individuals are more prone to respiratory disease after long-term exposure to high concentrations of PM_2.5_; additionally, the morbidity and mortality rates increase with exposure [8–10]. To protect public health, PM_2.5_ standards were proposed by US Environmental Protection Agency (USEPA) in 1997, with a short-term (24-h) standard value of 65 μg/m^3^ and a long-term (annual) standard value of 15 μg/m^3^ for PM_2.5_ [11]. Following several modifications derived from scientific research on the influence of PM_2.5_ on human health, the current short-term and long-term standards are 35 μg/m^3^ and 12 μg/m^3^, respectively [12].

In recent years, days with continuously high concentrations of PM_2.5_ have occurred frequently in central and eastern China. Early death of 1.23 million people and 25 million DALYs (disability-adjusted life years) lost are attributed tohigh concentrations of outdoor PM_2.5_ (2010) [3]. In January, 2013, approximately 600 million people living in a quarter of China's land area are at risk of PM_2.5_ [13]. Therefore, monitoring, predicting, and controlling PM_2.5_ is of great importance. On February 29, 2012, the Ministry of Environmental Protection of China unveiled a new revised "Ambient air quality standard" (GB3095-2012), which set the primary and secondary standards of PM_2.5_ concentrations for nature protection areas and residential districts respectively. The primary standards set an average of 35 μg/m^3^ for 24-hour period and 15 μg/m^3^ for one year, while the secondary standards are 75 μg/m^3^ as a 24-hour average and 35 μg/m^3^ as an annual average, respectively. With an annual average PM_2.5_ concentration of 133 μg/m^3^ in 2013, Xi'an City features one of the highest average concentrations of PM_2.5_ in China. Among 365 days in 2013, in total, there were 352 days and 259 days with concentrations greater than 35 μg/m^3^ and 75 μg/m^3^, respectively [14]. Approximately 6.5 million people in nine municipal districts, according to the sixth National Census of China in 2010, are often exposed to high PM_2.5_ concentrations. The health impact of this exposure is a serious concern. Research has shown that deaths caused by PM_2.5_ accounted for 1.6% of all deaths in Xi'an in 2010 [15]. Therefore, it is important to investigate the spatiotemporal and source patterns of PM_2.5_ concentrations in Xi'an City.

A series of models have been proposed to discern the relationships of pollutants and meteorological variables with PM_2.5_ concentrations. PM_2.5_ source apportionment models require the physical and chemical features of the chemical components and the quantitative determination theories [16, 17]. For these models, various chemical elements in PM_2.5_ are obtained with the help of chemical experiments by using the measurement of dozens of chemical elements (e.g., carbon), certain water-soluble ions, and several types of organic matter [18, 19].

Satellite data is a source of air quality variables for estimating PM_2.5_ concentrations. The Moderate Resolution Imaging Spectroradiometer (MODIS) aerosol optical thickness (AOT) product, an integral of aerosol extinction coefficients in the vertical direction from ground to top of atmosphere, is the relevant satellite remote sensing product that could be used to study variations in PM_2.5_ concentrations. MODIS product can be used to estimate and continuously monitor air quality variation and factors globally, while the ground-based measurements are only representatives at specific positions, primarily in urban regions, with a lack of spatial coverage and sparse monitoring data in rural areas [20]. The experiment conducted in Jefferson County, Alabama in 2002 demonstrated a strong relationship between AOT and PM_2.5_ with a linear correlation coefficient of 0.7 [21]. The strong correlation, especially under clear sky conditions with less than 40–50% relative humidity, was also validated in six other locations worldwide: Sydney, Delhi, Hong Kong, New York City, and Switzerland, where the linear correlation coefficient between bin-averaged AOT and PM_2.5_ concentration is 0.96 [22], and China [23–26]. Therefore, this product is substantiated by a series of experiments to demonstrate that AOT values have strong positive correlations with PM_2.5_ concentrations [27–31]. However, the relationship between AOT values and PM_2.5_ concentrations is also complex and non-linear, as they are both mixture that the ratios of components are different [32–36], and the relationship depends on multiple factors such as aerosol concentrations, relative humidity, and cloud coverage [22]. Seasonality appeared in the relationship between AOT and PM_2.5_ concentrations in the experiments conducted in the United States, Guangdong Province, and North China [37–39]. In northern China, the strong seasonal variation of the relationship is caused by the dominant aerosol types, implying that large dust particles and soil aerosols are the primary types in spring and summer, and smoke and soot aerosols are the dominant types in autumn and winter [39, 40].

In addition, gas-phase concentrations such as SO_2_ and NO_2_ could be the auxiliary variables for PM_2.5_ concentrations prediction, since they are the precursor gas contents of secondary ions of water-soluble inorganic salts [41], and can share a common emission source, be processed in the atmosphere and partition to the particulate-phase. In China, the mass concentrations of water-soluble inorganic salts and carbonaceous components make up more than 50% of PM_2.5_ [42–44]. Moreover, a few studies showed that the variations of PM_2.5_ concentrations were sensitive to meteorological variables [45, 46]. The integrated action of these variables, such as temperature, humidity and wind renders PM_2.5_ concentrations highly variable between different periods and regions.

A regular diurnal cycle with two maxima and two minima per day was obvious in the hourly PM_2.5_ concentrations of Xi'an City [47].The AOT values during particulate matter events including haze, dust storms, straw combustion, and fireworks displays, were twice as large as those of normal days [48]. Based on the emissions of organic and elemental carbon, gasoline engine exhaust, diesel exhaust and coal burning were the main contributors to PM_2.5_ in fall and winter in Xi’an City [49]. In 2010, the concentrations of organic and elemental carbon in PM_2.5_ in winter were 2.62 and 1.75 times higher than those in summer, primarily due to traffic emissions and adverse weather conditions [50]. Thus, the variation of PM_2.5_ in Xi’an city was related to the above emissions and meteorological conditions.

For these studies, linear regression analysis is utilized to determine the types of variables related to PM_2.5_. However, non-linear relationships between many variables render linear regression model descriptions inaccurate. Moreover, due to the complex and various chemical components of PM_2.5_ and the potential variables in different places and periods, their relationships and variation patterns can be inadequate in Xi'an City. For instance, the dominant species and their ratios are different for AOT in various seasons and areas, indicating that the relationships between AOT and PM_2.5_ concentration to be seasonal and non-linear. Additionally, the relationships (e.g., the effects, degrees and patterns) between meteorological variables and air quality indexes, and PM_2.5_ concentrations are complex. We expect that effects of temperature, wind directions and other variables are non-linear. Therefore, to understand the relationships among the multiple sources and various types of variables, we will have to use non-linear models.

The objective of this work is to explore statistical relationships between the variables and PM_2.5_ concentrations in Xi'an City in 2013, identify the primary variables, and to interpret the patterns of the relationships and understand the source patterns on the basis of the spatio-temporal evolution of PM_2.5_ concentrations. Ground-based monitoring data are provided by several ministries. Satellite remote sensing products will be used as well. Different types of data complement each other. Additionally, the comprehensive use of multi-source monitoring data is valuable to determine the values and causes of PM_2.5_ concentration levels in different periods and regions. Furthermore, a relationship between PM_2.5_ and its corresponding statistical variables such as pollutants and meteorological variables is constructed by applying a generalized additive model (GAM), which describes the non-linear relationship between variables and responses via nonparametric smoothing functions [51]. A series of experiments have demonstrated that a generalized additive model works better than a linear regression model for variables with non-linear relationships [52–54]. The generalized additive model approach is reliable and flexible for constructing relationships, exploring variables and making predictions [55–58].

## Materials and Methods

### Study area and sampling sites

The study area includes nine urban districts in Xi'an City, which is the capital city of Shaanxi Provence in China. Xi’an City, with an area of 3,581 square kilometers and a population of 6.5 million people, is located in the middle of the Yellow River’s Guanzhong Plain, with the Qinling Mountains to the south and the Weihe River to the north. The terrain features high elevations in the southeast and low elevations in the northwest. Days with continuous high concentration of PM_2.5_ occur frequently in central and eastern China in recent years. The annual average of PM_2.5_ concentration is 133 μg/m^3^ in Xi'an City, one of the cities with the most severe air pollution in China.

**Fig 1:**
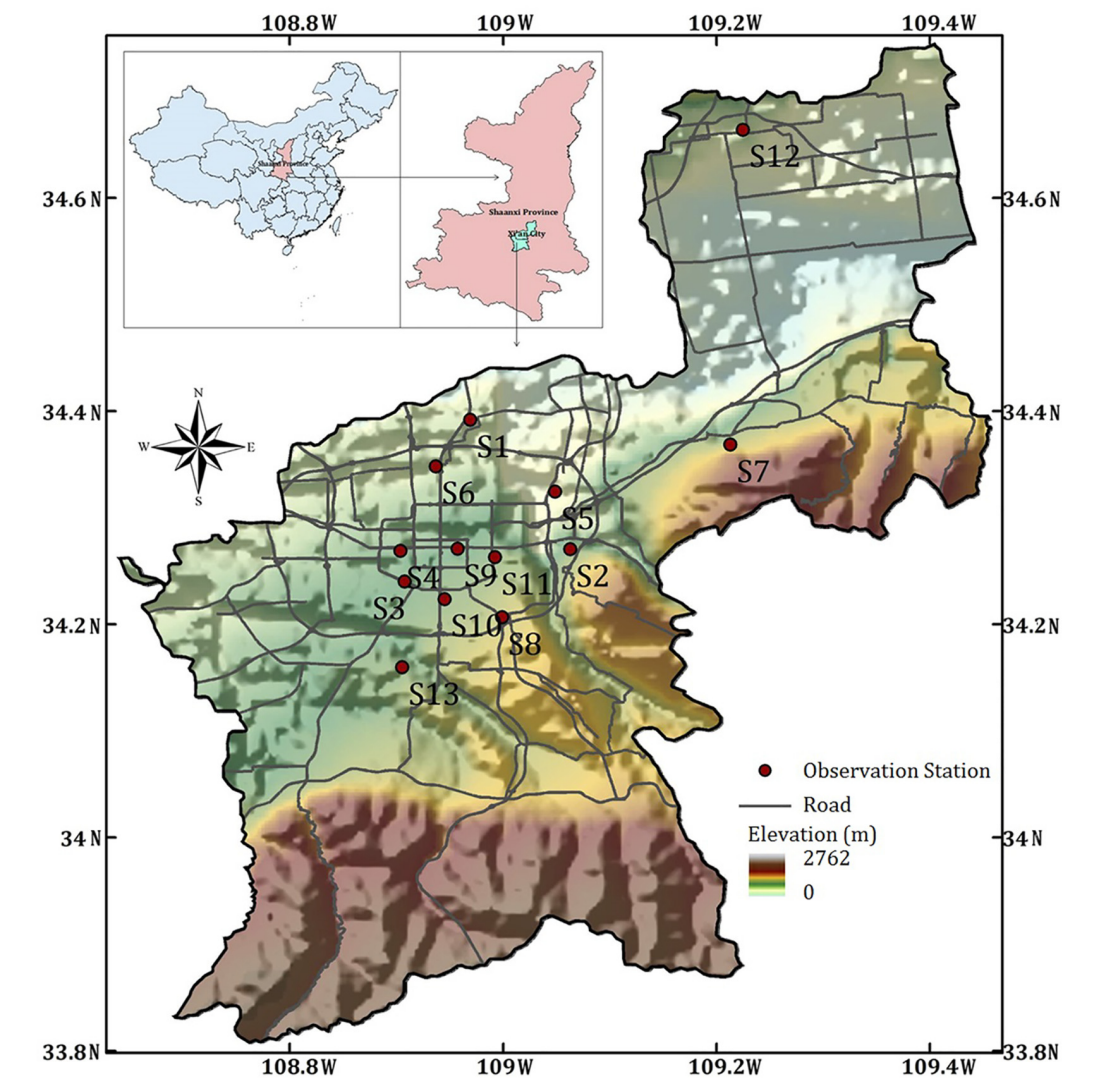
The location of Xi'an City in China and the distribution of 13 observation stations.

The research in this paper is based on daily PM_2.5_ concentrations, reported by the Xi'an environmental monitoring station [14] and obtained from 13 observation stations in Xi'an between January 1 and December 31, 2013. Fig 1 shows the location of Xi'an City in China and the distribution of the 13 observation stations on a digital elevation model (DEM) map. Stations 7 and 8 are located in mountainous regions with the elevations 500.2 m and 500.8 m respectively, and other stations are in plain area with the elevations ranging from 388.1 m (station 5) to 438.4 m (station 2). PM_2.5_ concentrations and four gas-phase concentrations (daily average concentrations of SO_2_, NO_2_, CO and O_3_) are measured daily at the 13 observation stations.

**Fig 2:**
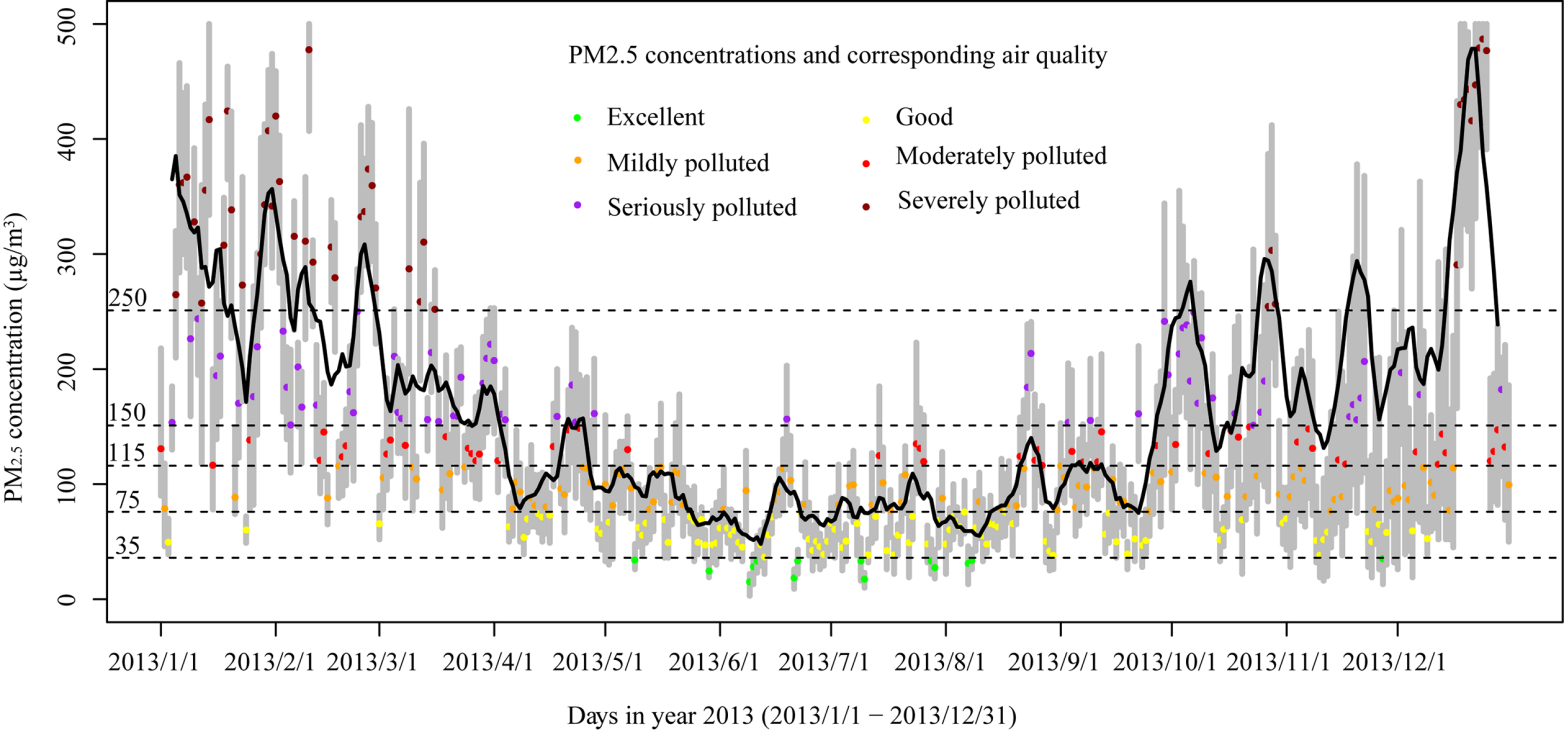
Time series of daily PM_2.5_ concentrations at 13 monitoring stations of Xi'an in 2013. The gray vertical lines indicate the range of daily PM_2.5_ concentrations at the 13 monitoring stations (from maximum to minimum), the colored dots are the average concentrations of each day (according to the defined colors in Table 1), and the black line illustrates the continuous 7-day average concentration.

In Table 1, the PM_2.5_ concentrations and air quality are classified according to the definitions in Technical Regulation on Ambient Air Quality Index (under review) (HJ 633–2012, China's environment protection standard). The air quality conditions can be presented with different colors. The time series of Fig 2 show the variation and seasonality of PM_2.5_ concentrations in Xi'an in 2013. We can see from the time series that excellent and good air qualities are primarily from May to August, and seriously polluted and severely polluted air qualities are from January to March and form October to December. Especially, severely polluted air qualities are in the whole winter (December, January, and February). Table 2 lists the summary statistics of the PM_2.5_ monitoring data (raw dataset is S1 Appendix). Missing PM_2.5_ concentrations data accounts for 10.7% (509/4745 data points). In other words, we use 89.3% of the raw data (4236 data points) to conduct the following experiment.

**Table 1:** Categories of air quality and corresponding PM_2.5_ concentrations.

Level of air quality index	Category of air quality	PM_2.5_ concentration (μg/m^3^)	Color
1	Excellent	0–35	Green
2	Good	36–75	Yellow
3	Mildly polluted	76–115	Orange
4	Moderately polluted	116–150	Red
5	Seriously polluted	151–250	Purple
6	Severely polluted	>250	Maroon

**Table 2:** Summary of data (except for wind direction) used to model the PM_2.5_ concentrations.

Variables	Units	Observation stations	No. of observations (missing percent, %)	Min	1st Qu.	Median	Mean	3rd Qu.	Max
PM_2.5_	μg/m^3^	13	4236 (10.73)	3	65	102	135.6	172	500
SO_2_	μg/m^3^	13	4137 (12.81)	0	18	32	38.83	55	135
NO_2_	μg/m^3^	13	3927 (17.24)	0	42	62	66.34	89	199
CO	mg/m^3^	13	4181 (11.89)	0	30	43	51.36	68	135
O_3_	μg/m^3^	13	4016 (15.36)	1	18	33	40.48	52	199
Temperature	°C	Xi'an City	365 (/)	-3.50	7.00	17.50	16.61	25.50	32.50
Wind scale	level	Xi'an City	365 (/)	3	3	3	3.038	3	5
AOT	/	13	4745 (/)	0.05	0.34	0.52	0.62	0.78	3.80

### Measurement of gas-phase concentrations and meteorological data

**Fig 3:**
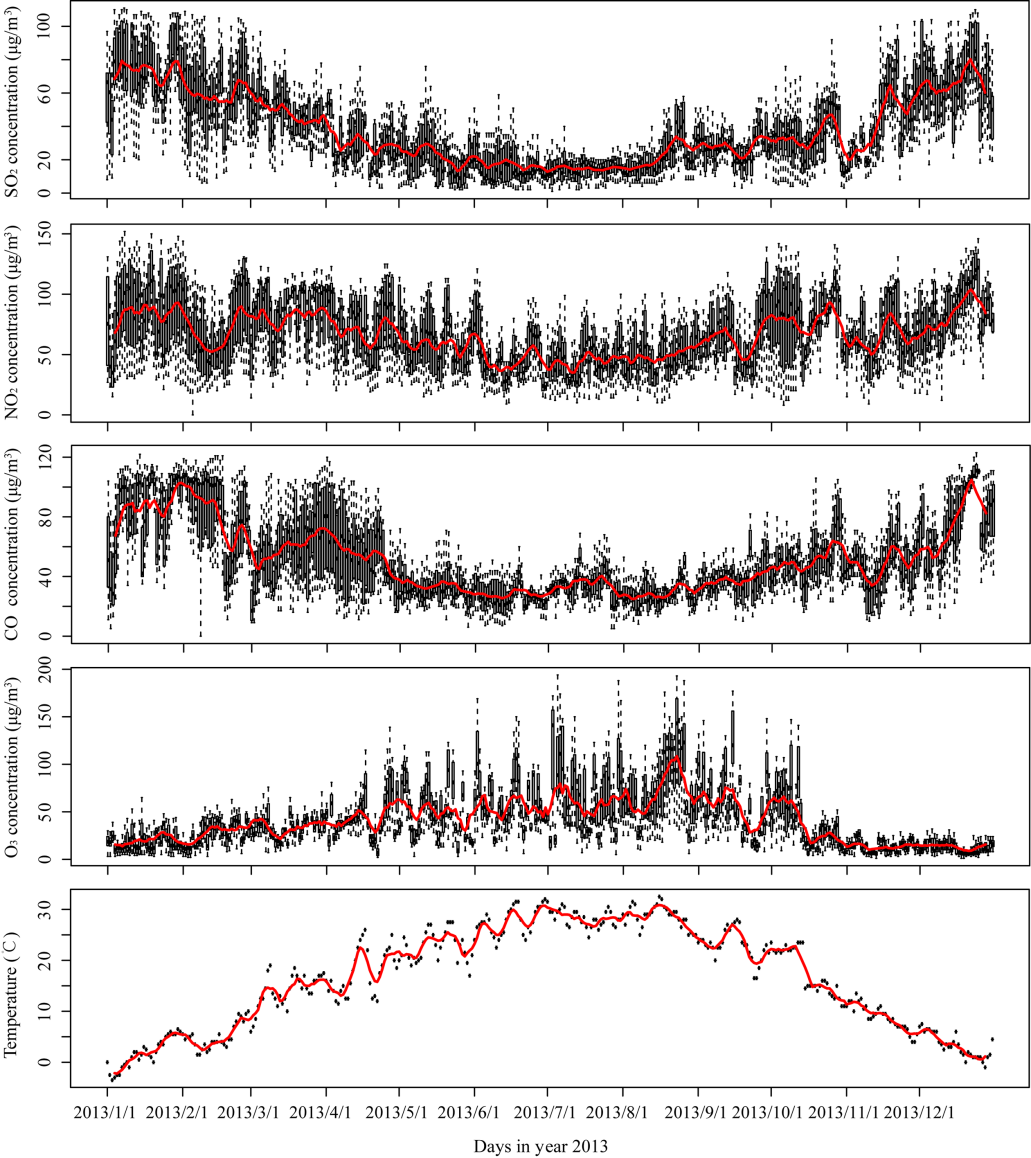
The time series of gas-phase concentrations (SO_2_, NO_2_, CO, and O_3_, μg/m^3^) and temperature (°C). The black vertical lines are the daily ranges of concentrations at 13 observation stations, and red line illustrates the continuous 7-day average concentration.

Daily average of gas-phase pollutant concentrations in the study area are also collected from the 13 monitoring stations of the Xi'an environmental monitoring network [14] in Xi'an City. These indices include daily average concentrations of SO_2_, NO_2_, CO and O_3_ measured at each station. Table 2 lists the summary statistics of these indices with a few missing observations. Similar to the time series of PM_2.5_ concentrations shown in Fig 2, the time series of four gas-phase concentrations and temperature in Fig 3 also show seasonality. The season with relatively high PM_2.5_ concentrations is winter. The pattern is identical with that of SO_2_ concentrations. While the largest values of NO_2_ and CO concentrations appear in both winter and spring, and the largest O_3_ concentrations occur in summer. Besides, the spatial differences of each variable change with the ranges of daily observations in 13 stations, as shown in the vertical black lines.

**Fig 4:**
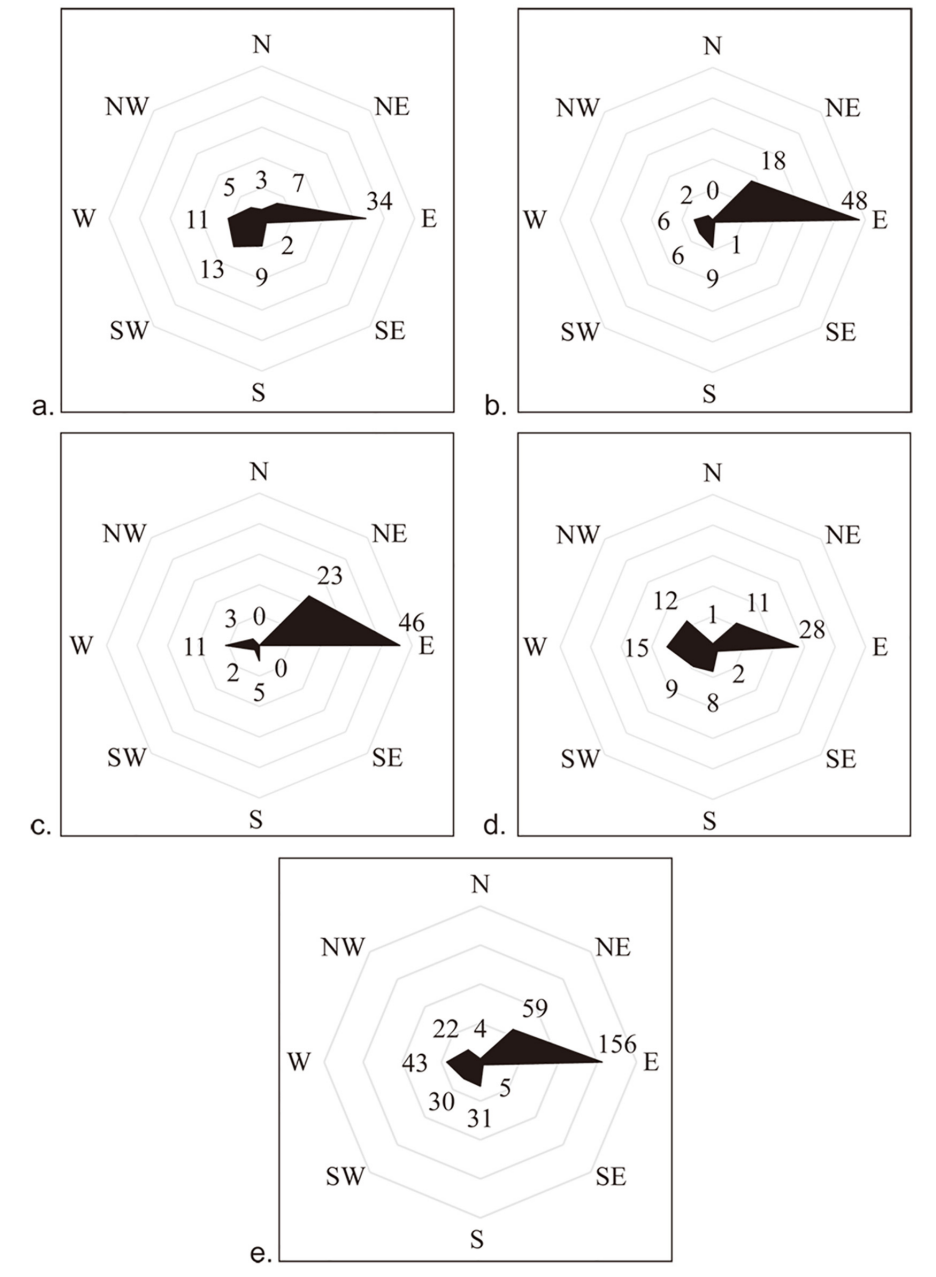
Number of days with the same wind directions in each season (a. spring; b. summer; c. autumn; d. winter) and the whole year (e.).

The meteorological data in study area consists of daily temperature (daily maximum temperature, minimum temperature and mean temperature), wind direction and wind scale in Xi'an City throughout 2013. The meteorological data are obtained from the China Meteorological Administration [59]. Table 2 lists the summary statistics of the daily mean temperature and wind scale, and Fig 4 shows the summaries of wind directions in each season (raw dataset is S1 Appendix). Because the actual observed meteorological data are invariant across Xi'an City for a given day [60] and the scale of the study area is not too large to discuss the variations in meteorological data, the application of a single weather observation to the whole region is reasonable.

A circular area with a radius of 14 km covers 11 observation stations (with stations S7 and S12 excluded). The distances between the circle’s center and the two excluded stations are about 25 km and 50 km, respectively. The correlation between the 47-year daily temperature series of Beijing, a city with the same monsoon climate and similar terrain as Xi'an, is significant (α = 0.01). For all the reference stations located within 340 km the correlation coefficients are larger than 0.94 [61]. Furthermore, the correlation coefficient between the daily temperatures of the two locations with a distance of 15.74 km is larger than 0.96, and the correlation between the stations with a distance of 55.51 km is larger than 0.94 [62]. Daily temperature variations in these 13 observation stations are small even constant in all seasons [63], so the use of single daily mean temperature for the whole study area is reasonable.

According to the Chinese National Standards (GB/T 19201–2006), wind speed is divided into a number of levels: level 3 (a breeze) defined at 3.4 m/s—5.4 m/s, level 4 (a soft breeze) at 5.5 m/s—7.9 m/s, and level 5 (a fresh breeze) at 8.0 m/s—10.7 m/s. Wind scale is the substitution variable for wind speed if wind speed is not available. Therefore, the wind-related variables are wind direction and wind scale. The wind scale for 96.4% of the days in 2013 (352/365) is at level 3 and the occurrence of levels 4 or 5 is rare. In 2013, easterly wind is the primary direction (42.7%, 156 days), as shown in Fig 4, and there are only 15 days with almost no wind. Wind direction and wind speed in Xi'an are primarily affected by the Pacific Ocean and Siberian air current. North-westerly wind is formed by the Siberian high-pressure system in autumn and winter, and south-westerly or south-easterly winds are related to the increasing or decreasing Pacific Ocean pressure in spring and summer [64–66]. Because the study area is much smaller than the area affected by air pressure, wind is usually constant among the observation sites in this study area. Ratios of days with easterly wind and north-easterly wind increase from 11.2% in spring to 18.1% in summer, but decrease from 18.9% in autumn to 10.7% in winter. Ratios of days with south-westerly wind, southerly wind, and westerly wind decrease from 9.0% in spring to 4.9% in autumn, but increase to 28.5% in winter.

### Aerosol optical thickness data

**Fig 5:**
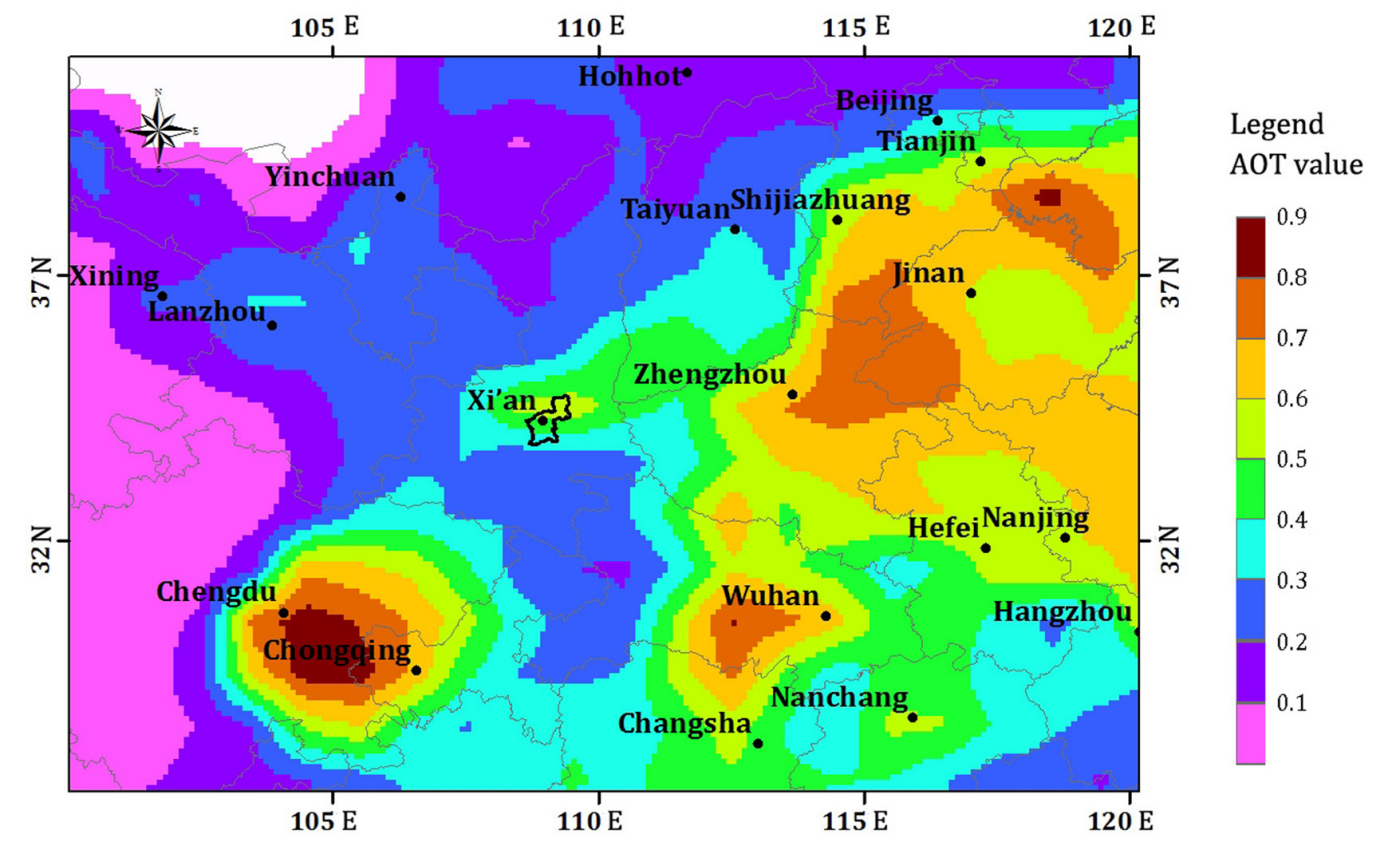
Spatial distribution of annual mean AOT in 2013 for Xi'an and its surrounding cities.

AOT, which is the integral of aerosol extinction coefficients in the vertical direction from the ground to the top of atmosphere, is an alternative satellite product to effectively predict PM_2.5_ concentrations. The strong correlation between AOT and PM_2.5_ concentrations has been documented by a series of studies in recent years [21, 23, 28]. The daily AOT (550 nm) data is a MODIS Terra Atmosphere level 3 product, downloaded from the Global Space Flight Center MODIS Level 1 and Atmosphere Archive and Distribution System Web [67]. The AOT values for the 13 observation stations are generated using the Ordinary Kriging method and the MODIS Terra Atmosphere level 3 product with a spatial resolution of 1°. Fig 5 shows the spatial distribution of the annual mean AOT (550 nm) obtained from the MODIS satellite data product for 2013 in Xi'an and its surrounding cities. This figure also shows that Xi'an City is one of the areas with relatively high AOT (550 nm) values.

### Modeling PM_2.5_ concentrations

**Fig 6:**
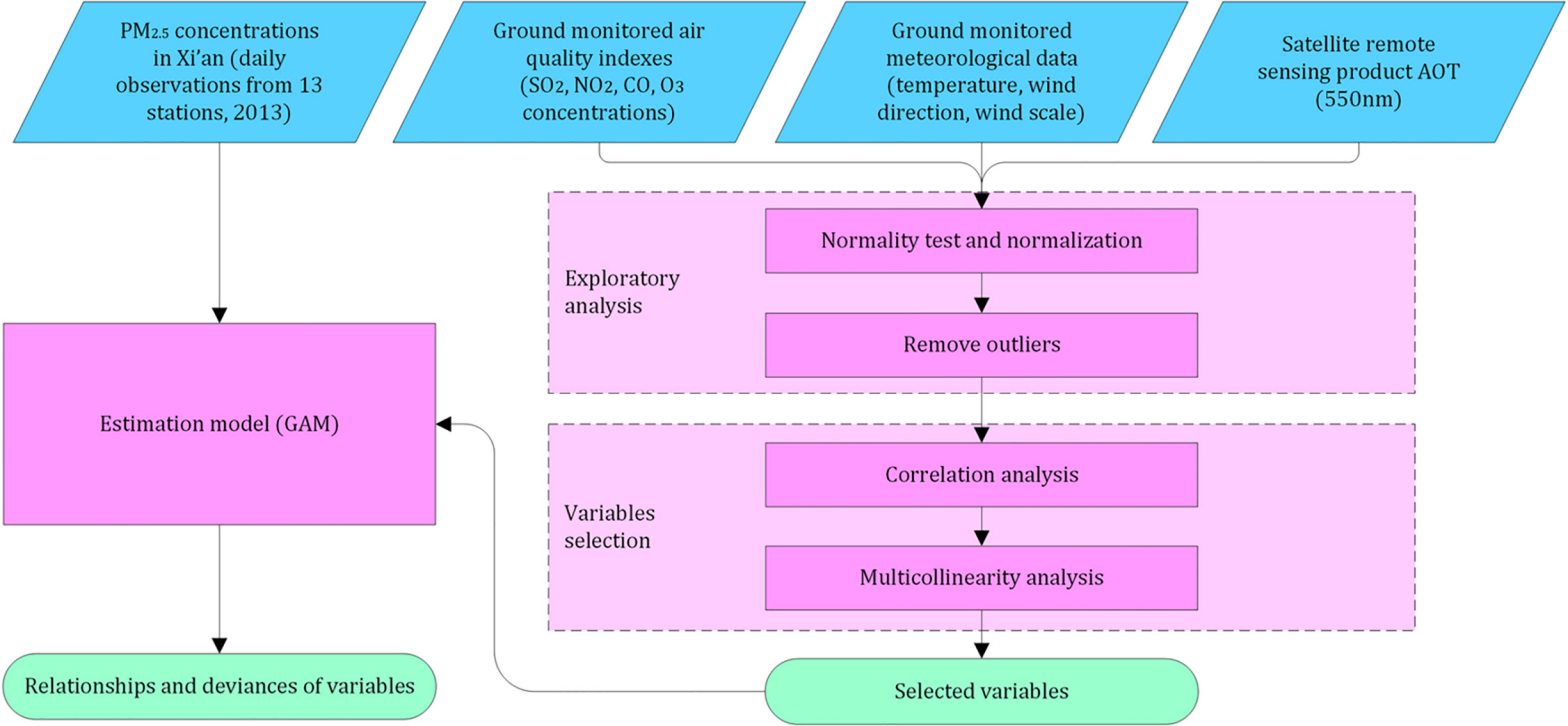
Schematic overview of the estimation experiment.

The flowchart of the experiment process is illustrated in Fig 6. The three major steps of the experiment are the exploratory analysis, variable selection and estimation model. The first step exploratory analysis aims at charactering PM_2.5_ concentration data, testing its statistical features, and removing the outliers. The target of the second step is to select the variables that could be statistically related to PM_2.5_ concentration for statistical models. Finally, the last step is to find potential non-linear relationships, to estimate model parameters and to quantify the variances of the potential variables.

### Exploratory analysis and variable selection

Exploratory analysis, based on the summary statistics of PM_2.5_ concentrations and the independent variables listed in Table 1, is a key step for processing raw data. Both dependent and independent variables are tested with Quantile-Quantile (QQ) plots and those that don’t follow a normal distribution are transformed to a normal distribution with natural logarithm. In this process, the variables PM_2.5_ concentration, O_3_ concentration, and AOT (550 nm) are transformed to the data with a normal distribution. Outliers, which generate anomalous analysis results, are removed by using the criteria. More specifically, a value with more than three times of the estimated standard deviation (> 3 σ) from the median is treated as an outlier from an assumed normal distribution [68]. As a result, a small number of outliers in PM_2.5_ concentrations data and certain other variables are removed.

The normalized variables without outliers are divided into the following groups: (a) the satellite-based data is AOT (550 nm) values; (b) the gas-phase concentrations are the daily average concentrations of SO_2_, NO_2_, CO and O_3_; (c) the meteorological variables of daily mean temperature, wind direction and wind scale; and (d) the spatial location variables of longitude and latitude. Temporal variables, such as days or seasons, are not considered, because the meteorological variables are physical reflections of temporal variables, and they are equivalent to the physical meanings of temporal variables in this particular case.

**Fig 7:**
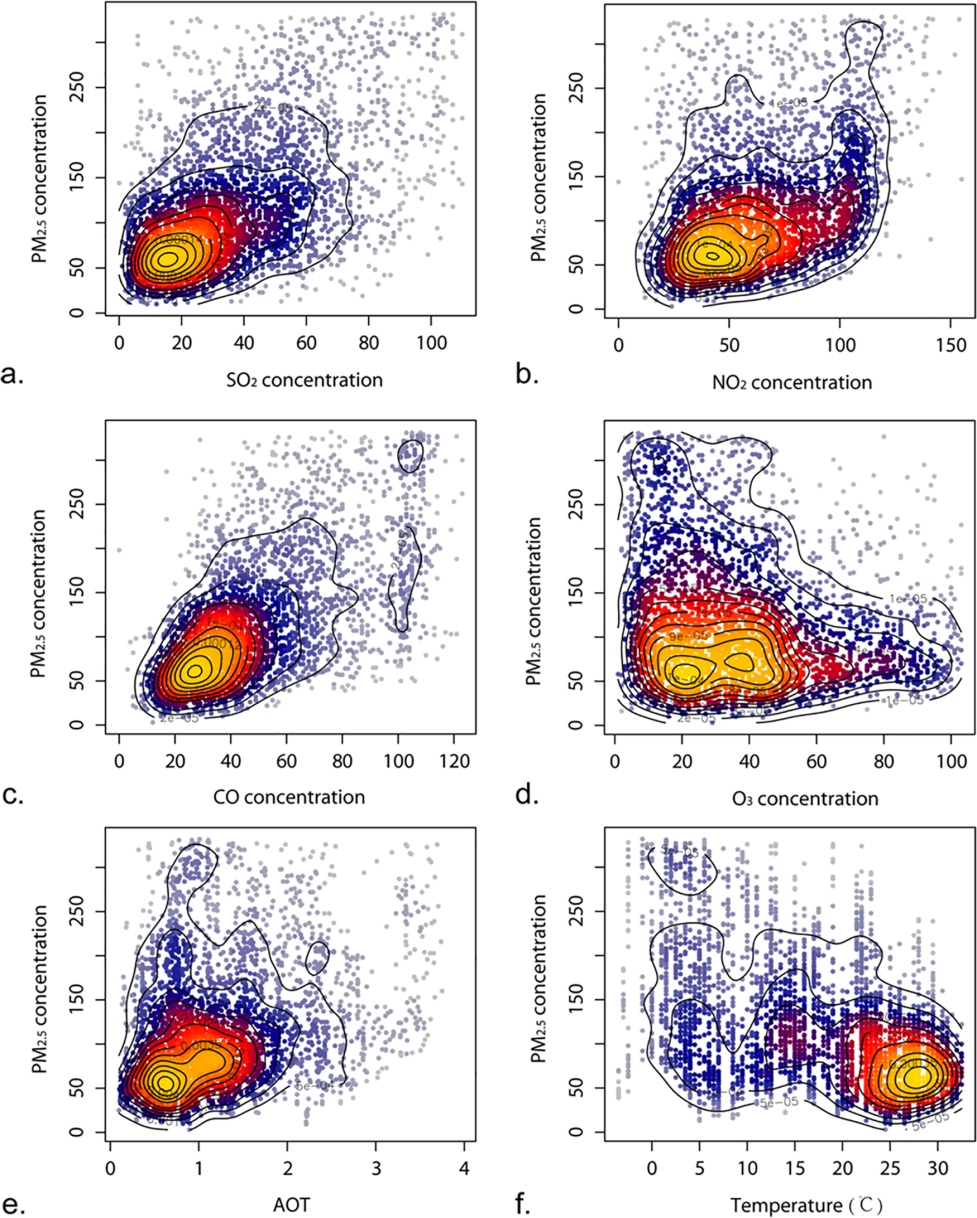
Scatter plots between PM_2.5_ concentration (μg/m^3^) and the variables gas-phase concentrations (SO_2_, NO_2_, CO, and O_3_, μg/m^3^), AOT values, and Temperature (°C).

**Fig 8:**
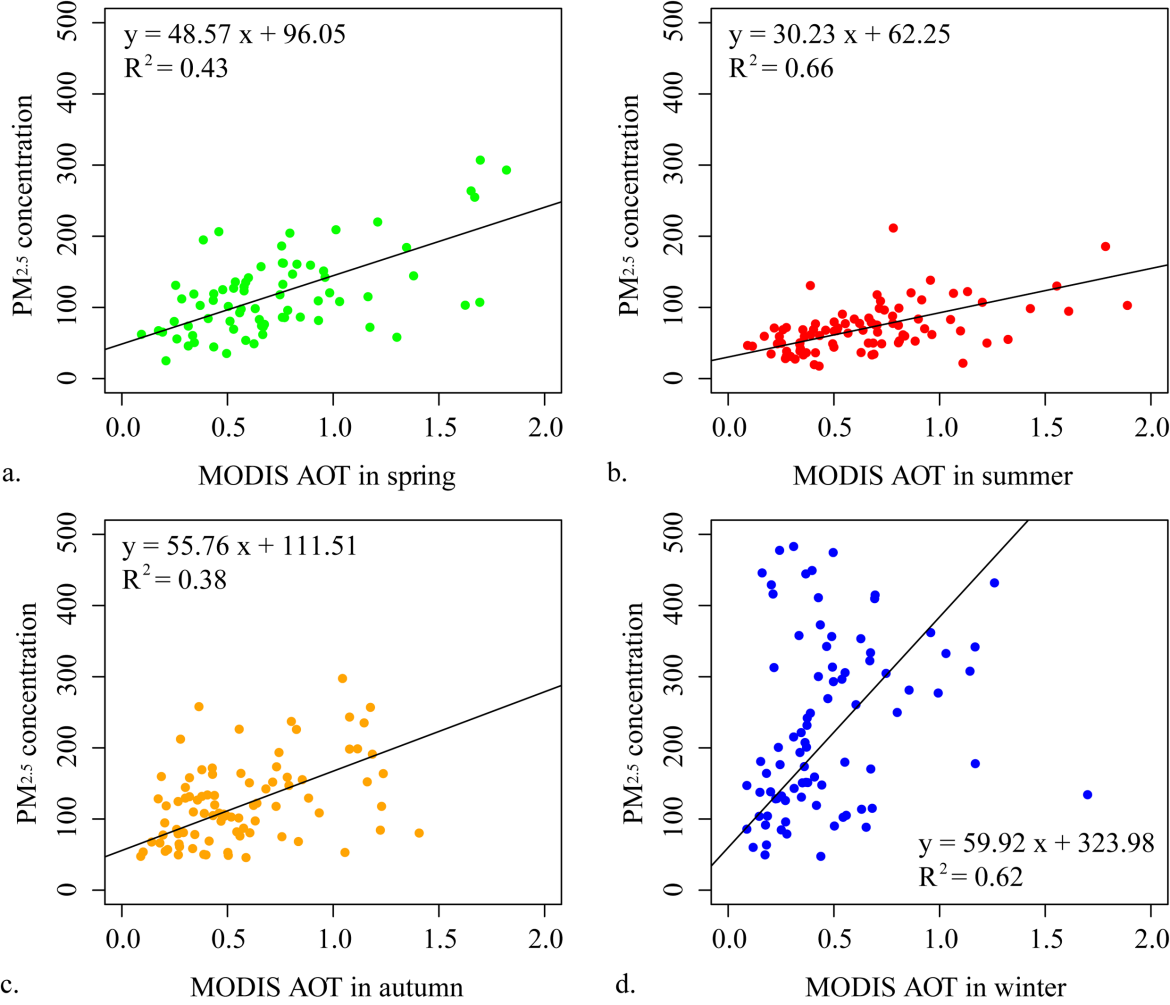
Linear regression functions between MODIS AOTs and PM_2.5_ concentrations (μg/m^3^) in different seasons.

Correlation analysis is conducted for these groups of variables to remove those without significant correlation with PM_2.5_ concentrations. Scatter plots between PM_2.5_ concentrations and the variables in group (a), (b), and temperature are used to depict and determine the linear or non-linear relationships. Fig 7 shows the relations between PM_2.5_ concentrations and the variable four gas-phase concentrations, AOT values, and daily mean temperature. The colors and contours represent the density of the scatters. It is clear from the figure that the relationships between PM_2.5_ concentration and concentrations of SO_2_ and CO are linear, but other relationships are complex and regarded as non-linear relationships. Further, in Fig 8, the seasonality between AOT (550 nm) and PM_2.5_ concentration is depicted with scatter plots and linear regression functions in the four seasons in Xi'an City. The regressions illustrate that the relationships between AOT (550 nm) and PM_2.5_ concentrations are distinct among the four seasons.

Pearson correlation coefficients between PM_2.5_ concentrations and the four air quality indices observed at the 13 stations each day were calculated. At this step, the non-linear relationships between the spatial locations of the observation stations and wind direction with PM_2.5_ concentrations must be considered. Additionally, the effect of AOT values on PM_2.5_ concentrations is seasonal and non-linear. Then, multicollinearity analysis is performed using variance inflation factors (VIFs) for the variables. The variables are divided into two groups, weakly correlated variables (VIF<10) and highly correlated variables (VIF≥10). One highly correlated variable is selected for combination with the weakly correlated variables, and VIFs are recalculated. This procedure is repeated, and the optimal combination of experimental variables is eventually chosen. In this experiment, the maximum values of VIFs are less than 4, so all VIFs are less than 10.

### Estimation model

A generalized additive model is used to construct the unknown statistical relationships, linear or non-linear, between PM_2.5_ concentration and the potential variables. In this work, the PM_2.5_ data form a continuous time series, but thirteen observation stations are not enough to perform spatial interpolation using geostatistics methods. Additionally, linear regression modeling is inappropriate when the dependent and independent variables are non-linearly related. The relationships between dependent variable, the pre-processed PM_2.5_ concentration, and independent variables are constructed via a generalized additive model under the assumption of normal distributions as follows:

$$y=\beta_{0}+\sum_{i=1}\beta_{i}x_{i}+\sum_{j=1}f_{j}(x_{j})+\sum_{k=1}f_{k}(x_{1,k},x_{2,k})+\varepsilon$$ (1)

where *y* is the dependent variable; *x*_*i*_ is a continuous linear variable; *x*_*j*_ is a continuous non-linear variable; *x*_*1*,*k*_, *x*_*2*,*k*_ are continuous non-linear variable-pairs; *β*_*0*_ and *β*_*i*_ are unknown coefficients; *f*_*j*_ and *f*_*k*_ are nonparametric smoothing functions between *y* and non-linear variables; and *ε* is the normal random error term (*ε*~*N*(0,σ^2^)).

In the model, wind variables are calculated as *w*_*c*_ ([wind scale]×cosine([wind direction])) and *w*_*s*_ ([wind scale]×sine([wind direction])). Additionally, the coordinates of the thirteen observation stations are used as variables. Because a few raw data points of certain variables were lost, the data used in the subsequent calculation are less than 4,745 lines (365 days × 13 observation stations). Therefore, data in 349 days (3067 lines) is available which is less 365 days of data because of missing data, and it becomes 333 days of data (2880 lines) when outliers are removed.

From the spatial perspective of geostatistics, generalized additive models are more flexible and reliable than spatial interpolation methods. To apply this method, a tensor product of longitude and latitude is constructed to describe the spatial distribution of PM_2.5_ concentrations, because the number of stations to monitor PM_2.5_ concentrations is only 13 and is not sufficient to estimate the PM_2.5_ concentrations of unknown coordinates via kriging interpolation. The reason is that too few observations will cause bias estimation [69]. However, estimates can be produced by taking longitude and latitude as variables and applying a bivariate smoothing function in a generalized additive model.

In this research, the mgcv package in the program R is used. The parameters of the smoothing functions are selected automatically by the generalized cross-validation (GCV) criterion due to its iterative approach, which improves calculation efficiency, which can help determine whether GCV score decrease when one variable is removed [70]. Three aspects are considered for model assessment: residual analysis for the model, comparison between the fitted values and observations, and fitness evaluation with the result comparison of generalized additive model to stepwise linear regression.

## Results and Discussion

In this research, satellite-based data AOT (550 nm), together with four gas-phase concentrations (SO_2_, NO_2_, CO, and O_3_), meteorological data (temperature, wind scale, and wind direction), and locations of observation stations (longitude and latitude), are treated as independent variables for constructing the relationships with dependent variable, namely, PM_2.5_ concentration, in Xi’an City. These variables reflect both air quality variations and meteorological conditions [71–73]; thus, the necessity of using both kinds of variables is affirmed. With the pre-process and consideration of each variable, the constructed generalized additive model is given as follows:

$$\begin{array}{l} \text{log}({\text{PM}}_{2.5})=3.97+4.17\times 10^{-3}\times SO_{2}+1.07\times 10^{-2}\times CO+s_{1}(NO_{2})+s_{2}(\text{log}(O_{3}))\\ \;+s_{3}\;(\text{log}(AOT))+s_{4}\;(Temperature)+t_{1}\;(w_{c},w_{s})+t_{2}\;(Lo,La)+\varepsilon \end{array}$$ (2)

where *s*_*i*_ (_*i*_ = 1, 2, 3, 4) is a univeariate smoothing function, the spline based smooth function; *t*_*j*_ (_*j*_ = 1, 2) is a bivariate smoothing function, the tensor product smooth function; *w*_*c*_ ([wind scale]×cosine([wind direction])) and *w*_*s*_ ([wind scale]×sine([wind direction])) denote the products of wind direction and wind scale, i.e., the substitution variable of wind speed; *Lo* and *La* are longitude and latitude, respectively.

**Fig 9:**
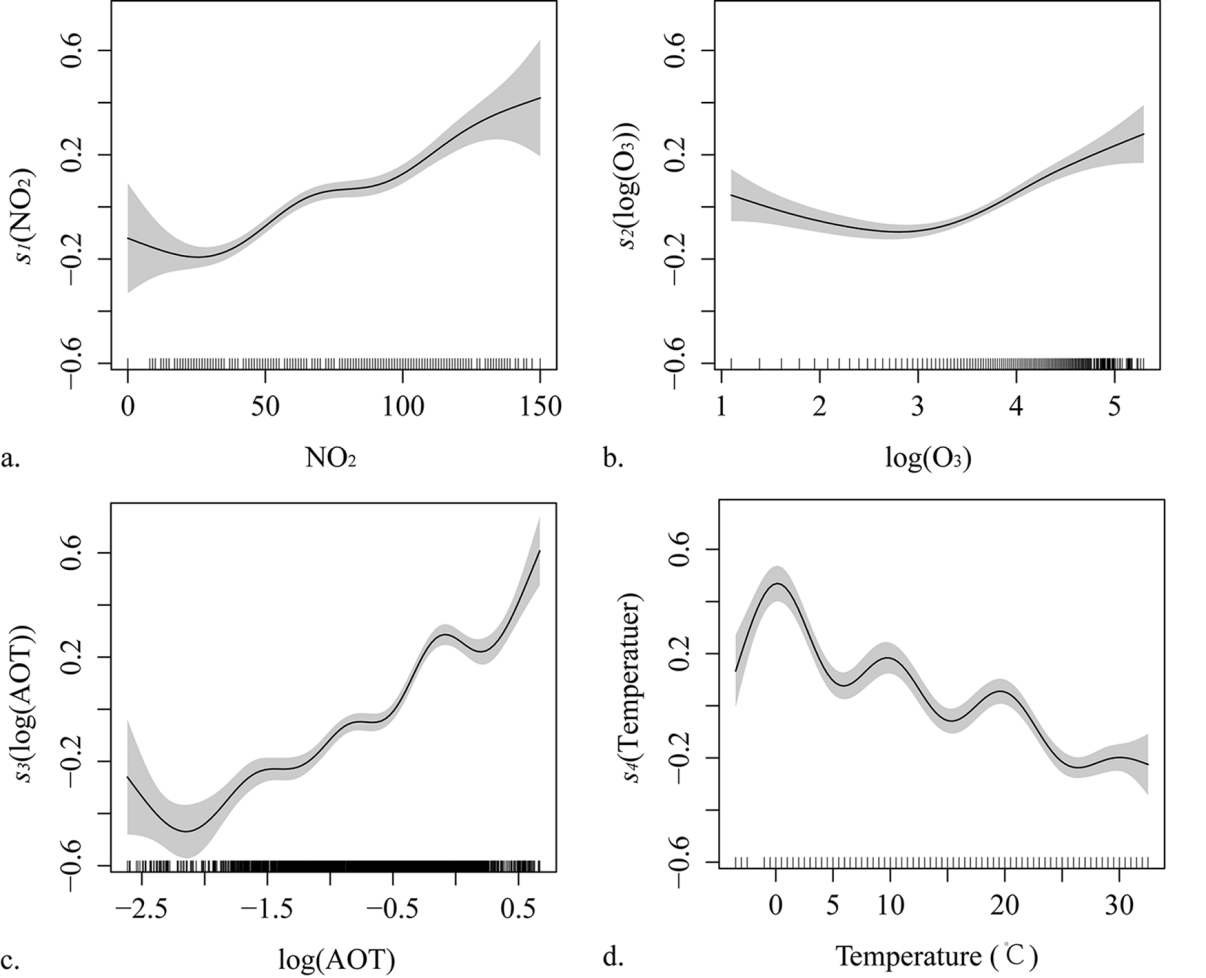
The effects of univariate smoothing functions for log(PM_2.5_) in the generalized additive model: (a) *s*_*1*_(NO_2_, μg/m^3^), (b) *s*_*2*_(log(O_3_), μg/m^3^), (c) *s*_*3*_(log(AOT)), and (d) *s*_*4*_(Temperature, °C). The gray shaded areas are the estimated 95% confidence intervals. The vertical lines adjacent to the x-axis indicate the presence of data.

**Fig 10:**
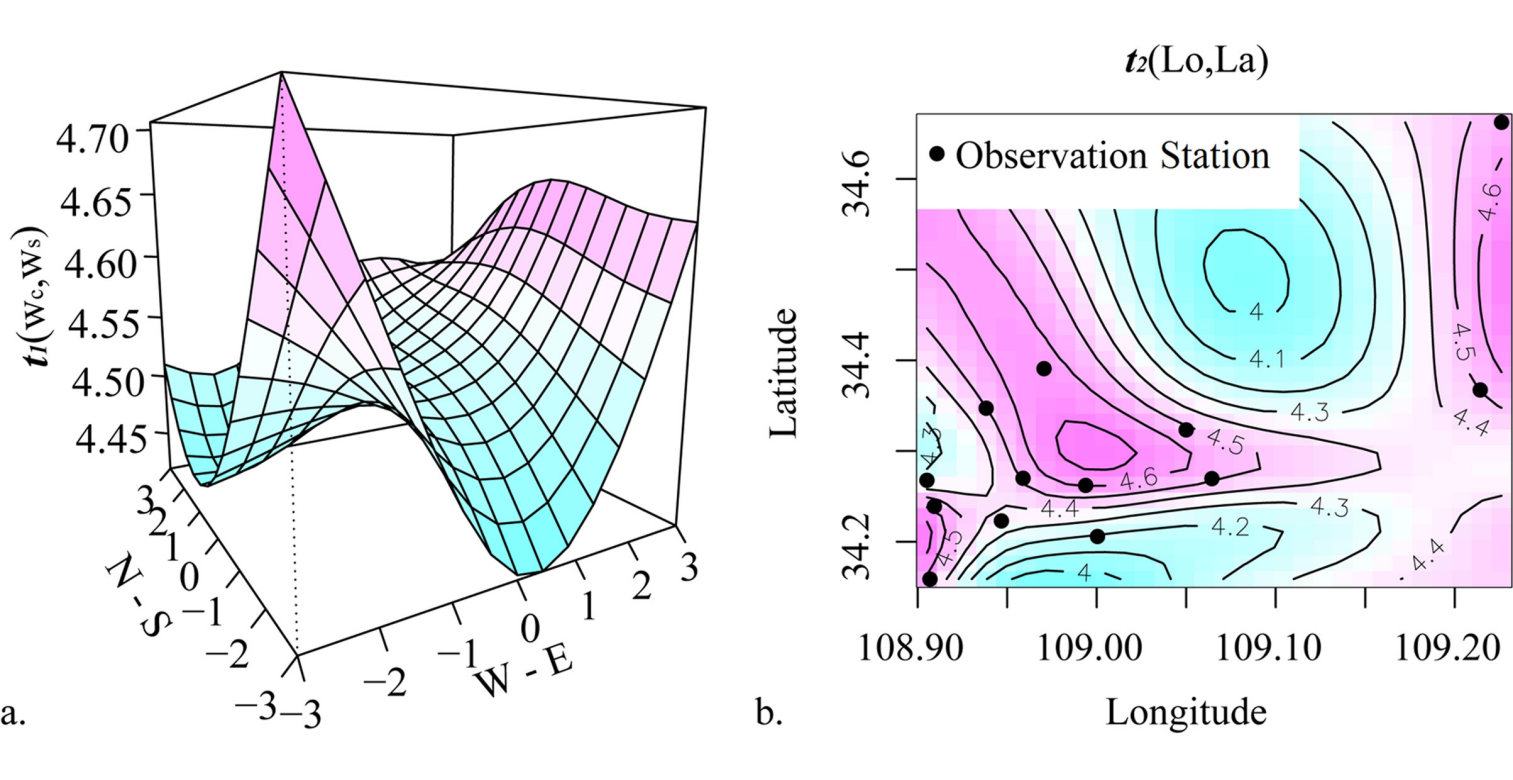
Bivariate smoothing functions of fitted components for log(PM_2.5_) in the generalized additive model. (**a**) Bivariate smoothing function for the wind components and the corresponding fitted values. (**b**) Bivariate smoothing function for longitude and latitude and the corresponding fitted values. The black dots represent the 13 observation stations that monitor PM_2.5_ concentrations.

The formula shows the concentrations of SO_2_ and CO are positively and linearly related with PM_2.5_ concentration, while other statistical relationships are non-linear. Different types of non-linear relationships can be expressed with these smoothing functions in the generalized additive model. Fig 9 shows the effects of the univariate smoothing functions on log(PM_2.5_ concentrations) in the model. In general, the smoothing function of NO_2_ concentration is an increasing non-linear function, but other three functions contain both increasing and reduction parts. When log(O_3_) is smaller than 3, the values are sparse and part of function is decreasing. On the contrary, when it is larger than 3, the values of log(O_3_) are dense and part of function is increasing. The function of log(AOT) rises with fluctuations though it declines at the small value part. Based on the smoothing function of daily mean temperature with fluctuation changes, the fitted PM_2.5_ values are highest when the daily mean temperature is 0°C. This result demonstrates that 0°C represents the suitable weather conditions for PM_2.5_ accumulation. Bivariate smoothing functions of fitted components for log(PM_2.5_ concentrations) in the generalized additive model are shown in Fig 10. In Fig 10A, vertical axis shows the bivariate smoothing function, *t*_*1*_(*w*_*c*_,*w*_*s*_), for the wind components and its corresponding fitted values. Southwest and east are the major wind directions affecting the variation of PM_2.5_ concentrations. In Fig 10B, the contour lines show the bivariate smoothing function (*t*_*2*_(*Lo*,*La*)) of location variable, i.e. longitude and latitude. In this figure, the black dots are the 13 observation stations monitoring PM_2.5_ concentrations. The location function suggests that PM_2.5_ concentrations in urban areas of Xi’an are higher than in suburbs.

**Fig 11:**
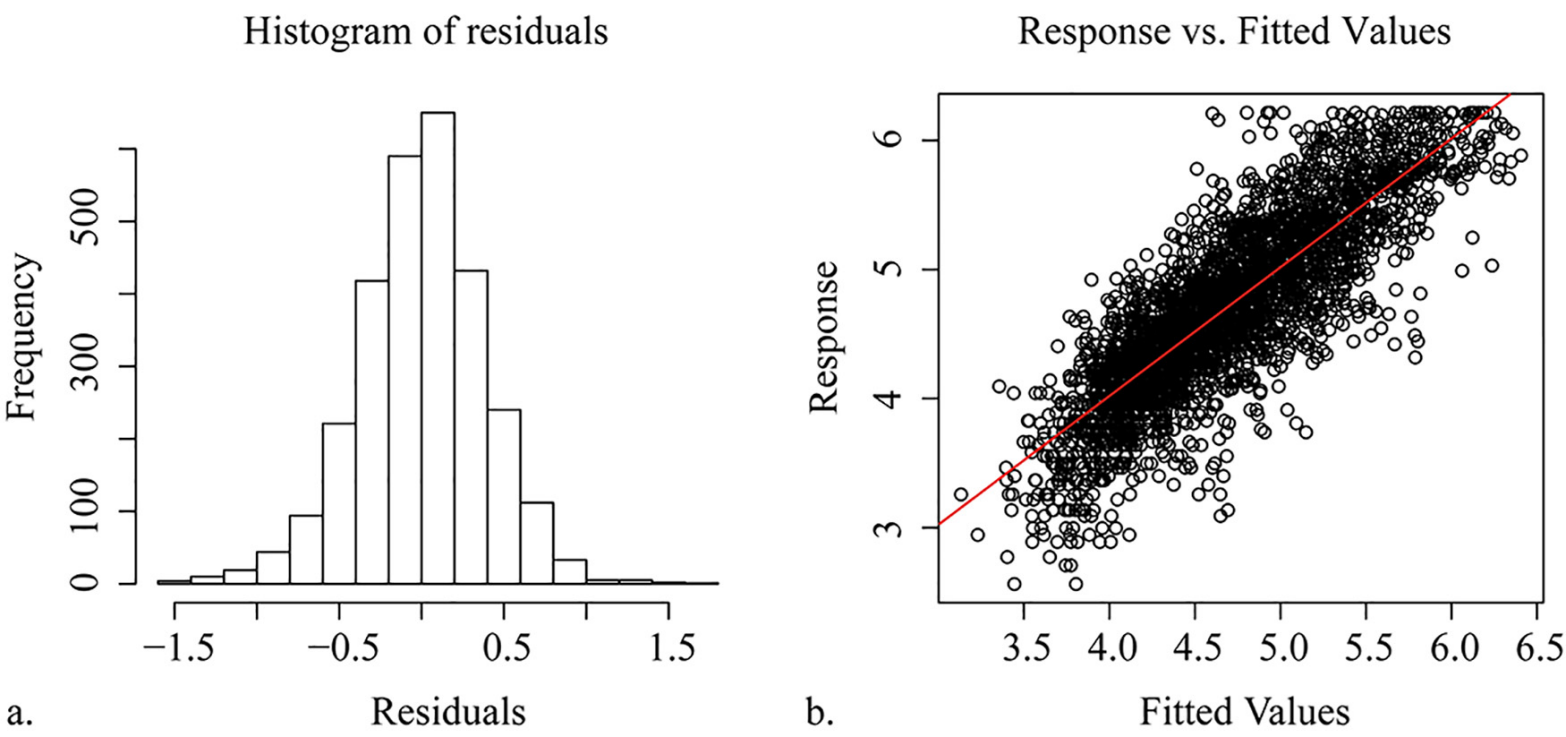
Evaluations of the model: (a) histogram of residuals and (b) fitted PM_2.5_ concentrations vs. observations.

The R^2^ value of the generalized additive model is 0.691, and the fitted residuals are normally distributed and not skewed (Fig 11). The model explains 69.50% of the total deviance in the PM_2.5_ concentrations data. To evaluate the model, a comparison between the estimated results and the results of a stepwise linear regression is considered, and evaluations of the fitted residuals and fitted values in this model are calculated. The fitting R^2^ value is 0.582 for the stepwise linear regression with the same variables and processed data after the exploratory analysis as those used in the generalized additive model. Therefore, the model in this paper is better than the stepwise linear model with the fitness improvement of 18.73%.

Since understanding the contributions of each variable to the total deviance is important, we calculate the contributions of the eight groups of variables and list the results in Table 3. Among these groups of variables, the linear variable CO concentration and log-transformed AOT (550 nm) values explain 20.65% and 19.54% of the deviance in PM_2.5_ concentrations, respectively. The total deviance that can be explained by gas-phase concentrations of SO_2_, NO_2_, and O_3_ is 10.88%. In this case, even half of this value is accounted for NO_2_ concentration. The other variables, with the decreasing contributions to the deviance, are the temperature, explaining 7.82% of deviance, the location, explaining 6.70% of deviance, and finally the wind, explaining 3.91% of deviance.

**Table 3:** Deviance explained by variables related to PM_2.5_ concentrations in Xi'an.

Variables	Deviance contribution
SO_2_ concentration	2.23%
NO_2_ concentration	5.86%
CO concentration	20.65%
O_3_ concentration	2.79%
Location (longitude, latitude)	6.70%
Wind direction (W-E, N-S) and scale	3.91%
Daily mean temperature	7.82%
Aerosol optical thickness	19.54%
Total deviance explained	69.50%

These results suggest that the PM_2.5_ concentrations in Xi'an City are most statistically related to carbon monoxide and aerosols. Carbon aerosols, a vital chemical component of PM_2.5_, and carbon monoxide share the same origin [74]. The common sources of carbon aerosols and carbon monoxide are likely due to traffic and other industrial emissions. This relationship supports the finding that CO concentrations and aerosols contribute most to the statistical relationships of PM_2.5_ concentrations in Xi'an. The two largest anthropogenic sources of carbon aerosols at the global scale are biomass combustion and fossil fuel combustion, while the major natural sources are plant emissions and natural fires [75]. The population density in study area is more than 1,800 people per square kilometer. In such a large city, human activities, such as fuel combustion, emission-generating vehicle operation and biomass burning, play an important role in forming large quantities of carbon aerosols. In Xi’an City, gasoline engine exhaust, diesel exhaust and coal burning accounted for the PM_2.5_ mass of 48.8%±10% in fall and 45.9%±7.5% in winter, respectively [49].

Apart from CO, the other three gas-phase concentrations, SO_2_, NO_2_, and O_3_, also contribute to PM_2.5_ concentration, though they appear to be less important than the above two variables, CO and aerosols. Sulfur dioxide and nitrogen dioxide are mainly released by traffic and industrial emissions in urban areas [76, 77]. Ozone is formed when oxygen molecules are combined with atoms released from nitrogen dioxide molecules in hot days [78–80]. It is formed primarily in urban areas from the end spring to early autumn, as shown in Fig 3. Therefore, the relationships between PM_2.5_ concentration and gas-phase concentrations could indicate that one of the important sources of PM_2.5_ is the traffic and industrial emissions.

The impact of daily mean temperature, a concrete expression of time, ranks after aerosols and gas-phase concentrations. Seasonality, with the lowest temperatures in winter, is the feature of temperature variation in Xi'an City. The period of highest PM_2.5_ concentrations occurs in winter because the weather conditions at 0°C are stable and favorable for PM_2.5_ accumulation.

In addition, 6.70% of the deviance is explained by the coordinates of the observation stations. The result of location non-linear function in Fig 10 shows that the PM_2.5_ concentrations in the urban areas are higher than those of suburbs in Xi'an spatially. This result confirms that AOT product is more significant for the global estimation of PM_2.5_ concentration, especially in the rural areas, because PM_2.5_ concentrations are different for urban and rural areas and because most of the ground-based measurements only provide the monitoring concentrations in urban areas.

Wind direction and wind scale have limited but still important influences on the concentration of PM_2.5_. Southwesterly wind and easterly wind contribute to higher concentrations relative to the other wind directions. According to this dataset and previous records of the climate in Xi'an, southwesterly wind and easterly wind primarily occur in the spring and summer respectively as a result of increasing and decreasing Pacific Ocean air pressure systems. Given the topographic conditions of Xi'an City and the distribution of its surrounding AOT (550 nm) values, easterly wind enables PM_2.5_ in the eastern areas with relatively high AOT (550 nm) values to spread to Xi'an City because the eastern area is connected with other AOT (550 nm) concentrated areas through the channel shown in Fig 5. Thus, wind has a weak influence on the variation in PM_2.5_ concentrations. The reasons are that southwesterly and easterly winds play the most important role in increasing concentrations. Nevertheless, the concentrations are relatively low in the seasons when they frequently occur. However, during winter and autumn, northwesterly winds have a more limited effect than southwesterly and easterly winds. The resuspension of dust is enhanced in spring and summer due to higher wind speeds and is reduced in winter when the soil is damp, which limits the release of soil-derived particles [81–83]. Thus, the relatively high concentrations are mainly produced locally in winter and autumn instead of coming from other regions through the channel mentioned above. From the above discussion, we may conclude that the high concentrations in winter primarily come from local emissions in Xi’an, such as home heating and the imperfect combustion of fuel. Whereas in spring and summer, the higher concentrations are possibly related to the concentrations of neighboring areas.

Still, there are certain limitations in the experiment. To ensure the strictness and accuracy of the model and its calculating process, a normal distribution is assumed in the model, which leads to normalizing and outlier removal for large amounts of data. In addition, wind scale is used in the model as an approximation of wind speed because of the lack of wind speed data. Using a single meteorological monitoring site for the whole study area is limited, for wind direction might vary at different local places, though the trend in large spatial range is consistent. Because the focus of this article is on modeling the non-linear regression, a variety of variables are not introduced into the model as independent variables. Certain natural attributes, such as highly spatially resolved elevation, land use and even social attributes (e.g., population density) [84], could be treated as variables related to the variation in PM_2.5_ concentrations on account of previous authoritative studies and strict experiments.

## Conclusion

A generalized additive model was constructed and used to estimate PM_2.5_ concentrations in Xi'an City. Various variables from multiple monitoring data sources have been used, including satellite remote sensing product AOT, ground-based gas-phase concentrations, and meteorological monitoring data. The observed data are significant for constructing the statistical model. The model can explain 69.50% (R^2^ = 0.691) of the total deviance in the PM_2.5_ concentrations. The most significant variables, CO and AOT (550 nm), account for 20.65% and 19.54% of the deviance, respectively, indicating that the variation in PM_2.5_ concentrations is strongly correlated with CO and AOT. The total deviance that can be explained by three other gas-phase concentrations, SO_2_, NO_2_, and O_3_ is 10.88%. These results demonstrate biomass and fossil fuel combustion, likely produced by traffic and other industrial emissions, are the primary source of PM_2.5_ in Xi'an City. Additionally, the results have shown that temperature influences the concentrations, especially in winter when source emissions increase due to home heating and favorable temperature conditions of around 0°C for PM_2.5_ accumulation. PM_2.5_ is more likely to be accumulated in urban areas than in suburbs, which also affirms that satellite-based data is essential for predicting PM_2.5,_ since most of the ground observation stations are located in urban areas. Wind has a weaker influence in winter and autumn than in spring and summer; thus, PM_2.5_ is primarily produced locally in the cold seasons from heating and fuel combustion but is spread from other nearby areas in the warm seasons.

## Supporting Information

S1 Appendix Raw dataset of PM_2.5_ concentration, gas-phase concentrations (SO_2_, NO_2_, CO, and O_3_), AOT, temperature, wind direction, wind scale and locations of 13 observation stations (longitude and latitude). (XLSX)
